# Treg and Oxidative Stress Biomarker Profiles in Long COVID and Myalgic Encephalomyelitis/Chronic Fatigue Syndrome

**DOI:** 10.3390/ijms27219422

**Published:** 2026-10-22

**Authors:** Yana Todorova, Radostina Dimitrova, Teodora Kalfova, Steliyan Petrov, Martina Bozhkova, Mariya Ivanovska, Dobrina Dudova, Mariana Murdjeva, Hristo Taskov, Michael Maes, Maria Nikolova

**Affiliations:** 1 Center of Competence Immunopathogen, National Center of Infectious and Parasitic Diseases (NCIPD), 1504 Sofia, Bulgaria; todorova_yana@ncipd.org (Y.T.); rdimitrova@ncipd.org (R.D.); 2 Research and Innovation Program for the Development of MU-PLOVDIV (SRIPD-MUP), Creation of a Network of Research Higher Schools, National Plan for Recovery and Sustainability, EuropeanUnion–NextGenerationEU, Medical University of Plovdiv, 4002 Plovdiv, Bulgaria; hristotaskov@gmail.com (H.T.); dr.michaelmaes@hotmail.com (M.M.); 3 Department of Medical Microbiology and Immunology “Elissay Yanev”, Medical University of Plovdiv, 4002 Plovdiv, Bulgaria; teodora.kalfova@mu-plovdiv.bg (T.K.); steliyan.petrov@mu-plovdiv.bg (S.P.); martina.bozhkova@mu-plovdiv.bg (M.B.); mariya.ivanovska@mu-plovdiv.bg (M.I.); dobrina.dudova@mu-plovdiv.bg (D.D.); 4 Research Institute, Medical University of Plovdiv, 4002 Plovdiv, Bulgaria; 5 Department of Microbiology and Virology, Medical University of Pleven, 5800 Pleven, Bulgaria; mmurdjeva@yahoo.com; 6 Institute of Innovation and Smart Technology, University of Telecommunication and Posts, 1700 Sofia, Bulgaria; 7 Sichuan Provincial Center for Mental Health, Sichuan Provincial People’s Hospital, School of Medicine, University of Electronic Science and Technology of China, Chengdu 610072, China; 8 Key Laboratory of Psychosomatic Medicine, Chinese Academy of Medical Sciences, Chengdu 610072, China; 9 Department of Psychiatry, Medical University of Plovdiv, 4002 Plovdiv, Bulgaria

**Keywords:** long COVID, ME/CFS, Th17, Treg, MDA, GPX1

## Abstract

Chronic immune dysregulation is the possible pathophysiological mechanism in myalgic encephalomyelitis/chronic fatigue syndrome (ME/CFS) and long coronavirus infectious disease (long COVID, LC). However, a detailed characterization of regulatory and effector T-cell subsets in these conditions remains limited. To assess and compare T-cell subsets in relation to serum biomarkers of chronic activation and oxidative/nitrosative stress in an attempt to differentiate between LC and ME/CFS patients. Peripheral blood mononuclear cells (PMNC) from LC patients (*n* = 89), ME/CFS patients (*n* = 125), and healthy controls (HC, *n* = 80) were analyzed using multiparameter flow cytometry. The frequencies of naïve (CD45RA+), effector (CD45RA-), CD31+, CD39+, CD73+, GARP+, PI16+, CD15s/CD161+, and CD147+ Treg subsets were determined in relation to CD4+ and CD8+ T-cell differentiation, activation, and effector subsets. Catalase (CAT), superoxide dismutase 1 (SOD1), glutathione peroxidase 1 (GPX1), malondialdehyde (MDA), nitric oxide synthase 1 and 3 (NOS1, NOS3), zonulin, lipopolysaccharide (LPS), immunoglobulin G (IgG) against lipopolysaccharide (IgG-LPS), Toll-like receptor 4 (TLR4), indoleamine 2,3-dioxygenase (IDO), and Dickkopf-related protein 1 (DKK1) were measured in serum by ELISA. Clinical symptom scores (HAMA, HAMD, FF, dePol) were additionally assessed. The immune-serum biomarkers distinguishing LC, ME/CFS, and HC comprised Th17, CD15s+CD161−eTreg, and CD161+ Treg. The five-marker signature retained significance after adjustment for age, BMI, and sex. Altered T-cell subsets were strongly associated with markers of oxidative (GPX1) and nitrosative (NOS3) stress but not directly with symptom severity. ME/CFS showed a strong separation from HC across Random Forest (AUC = 0.907) and logistic regression (AUC = 0.924). The modest distinction of LC from HC (AUC = 0.727 and 0.577 respectively) possibly reflected important endotypic diversity, though differences in disease duration, vaccination history, biomarker selection, and other cohort characteristics might add to it. MDA was an independent predictor for three-class classification, ME/CFS vs. HC, and LC vs. HC pairwise comparisons, while GPX1 discriminated between ME/CFS and LC. In spite of shared biomarkers of oxidative stress and T-cell response dysregulation, our results support the hypothesis that LC and ME/CFS differ in the extent of cellular damage and the exhaustion of compensatory mechanisms.

## 1. Introduction

Myalgic encephalomyelitis/chronic fatigue syndrome (ME/CFS) and long COVID (LC) represent two of the most prevalent, disabling, and poorly understood post-viral syndromes of the modern era. Both conditions are associated with high rates of occupational disability, social isolation, and significant deterioration in health-related quality of life, yet neither currently has a validated biological diagnostic marker or an approved disease-modifying therapy [1].

Severe acute respiratory syndrome coronavirus 2 (SARS-CoV-2) is the specific cause of LC, while a variety of infectious or non-infectious stressors have been proposed to trigger ME/CFS. On the other hand, the clinical presentation of LC may differ importantly in relation to acute infection severity, vaccination status or pandemic wave [2]. Despite this heterogeneity, the two conditions share a convergent clinical phenotype: profound, non-refreshing fatigue; post-exertional malaise; orthostatic intolerance in the form of postural orthostatic tachycardia syndrome or related autonomic dysfunction; unrefreshing sleep; and cognitive impairment termed ‘brain fog’ [3,4]. In fact, a significant proportion of LC patients fulfill diagnostic criteria for ME/CFS [5].

At the biological level, ME/CFS and LC share a range of immunological, metabolic, and neuroendocrine abnormalities that suggest disruption of fundamental homeostatic mechanisms [3,6]. Persistent immune activation—characterized by aberrant cytokine profiles, CD8+ T-cell exhaustion, natural killer (NK) cell cytotoxic impairment, and evidence of autoantibody generation—has been documented in both conditions [3,7,8]. Shared immune exhaustion gene expression profiles in peripheral blood mononuclear cells (PBMCs) from ME/CFS and LC patients further support the existence of convergent immune pathology [9]. A unifying hypothesis is that an initial infectious trigger sets in motion a self-perpetuating immune dysregulation [6,10].

Antigen-driven T-cell differentiation provides effector subsets to efficiently remove pathogens, long-living memory subsets to perpetuate immunity, and regulatory subsets to dampen excessive immune activation [9]. The balance between effector, memory and regulatory subsets is critical for a protective immune response to infection. Effector Th cell subsets producing different cytokines are involved in protective immunity to different pathogens, including Th1, Th2, Th17 and related Th22, T follicular helper (Tfh) and ThG cell clones [9,10,11,12]. Naïve CD4 T cells can also differentiate into regulatory T cells (Tregs), defined by high expression of the master transcription factor Forkhead Box Protein P3 (FOXP3), along with high expression of CD25 (IL-2 receptor alpha-chain) and low/absent CD127 (IL-7 receptor alpha-chain). Tregs exert non-specific suppression on effector immune responses through multiple non-redundant mechanisms [13] and are indispensable for the maintenance of peripheral immune tolerance and prevention of autoimmunity [14]. In addition, Tregs modulate innate immune cell activation—including microglia, mast cells, and innate lymphoid cells—and interact bidirectionally with the autonomic nervous system (ANS) and the hypothalamic–pituitary–adrenal (HPA) axis, placing them at the intersection of immune and neuroendocrine regulation [15]. Therefore, among the immunoregulatory mechanisms whose dysfunction may underpin the persistent immune dysregulation observed in ME/CFS and LC, regulatory T cells (Tregs) appear to be of particular mechanistic importance.

Importantly, Th differentiation profiles are not epigenetically fixed. Under conditions of chronic or intense activation, the FoxP3 locus can be methylated, and Tregs converted to pro-inflammatory ‘ex-Treg’ phenotypes with Th17-like properties [16]. Moreover, Tregs are not a homogeneous population but comprise multiple subsets with differential contributions to immune regulation [17]. Peripherally induced Tregs are major down-regulators of pathogen-specific immune responses, and their malfunction has been associated with persistent immune activation in HIV and other chronic infections [9]. Naïve or resting (CD45RA+FoxP3^low^) vs. effector (CD45RA−FoxP3^high^) fractions carry distinct functional properties and disease associations [17]. Among activated effector Treg subsets, the associated expression of CD73 and CD39 enzymes generates immunosuppressive adenosine and characterizes a highly suppressive Treg subset implicated in autoimmune and post-viral pathology [18]. Peptidase inhibitor PI-16 identifies a memory Treg subset that can migrate to sites of inflammation and regulate pro-inflammatory responses [19]. Glycoprotein A repetitions predominant (GARP) is a transmembrane protein on the surface of Tregs, promoting the release of active TGF-β to suppress immune responses [20]. CD147/Basigin maintains Foxp3 protein levels and Treg inhibitory function, making it crucial for preventing excessive inflammation [21]. Highly suppressive effector Tregs are CD15s (Sialyl Lewis X antigen), while the CD15s-CD161+ phenotype suggests a distinct, more immature subset with increased “plasticity”, CD161 itself being a marker of Treg subsets capable of producing proinflammatory cytokines [22,23]. CD31 has a critical role in modulating Treg migration and metabolism, particularly through the regulation of glucose metabolism [24].

Current published results are largely restricted to the analysis of bulk Tregs without systematic subpopulation characterization. In ME/CFS, altered circulating Tregs have been frequently reported, though the direction and magnitude of these changes vary across cohorts [25,26]. Sepúlveda et al. [27] proposed that in ME/CFS, Tregs suppress anti-viral effector responses while simultaneously failing to resolve chronic immune activation—a paradox that has not been resolved by functional studies. In LC, deep immunophenotyping by Yin, Peluso, and Roan et al. [28] identified elevated total Treg frequencies alongside exhausted SARS-CoV-2-specific CD8+ T cells eight months post-infection, consistent with chronic antigenic stimulation driving compensatory regulatory expansion. Galan et al. [29] demonstrated significant reductions in circulating Treg proportions and CD39+Treg frequencies specifically in female LC patients meeting ME/CFS diagnostic criteria. None of these studies comprehensively characterized the full spectrum of Treg subpopulations. Moreover, the relationship between subpopulation-specific Treg abnormalities and the clinical heterogeneity of these conditions has not been explored.

The present study was designed to address the above gaps through comprehensive characterization of T-cell immunometabolic biomarkers in well-defined cohorts of ME/CFS and LC patients compared to healthy controls. We aimed to quantify the frequencies of phenotypically defined Treg subsets (CD45RA−, CD39+, CD73+ GARP+ PI16+, CD15s-CD161+, CD31+, CD147+ and GARP+) in peripheral blood, evaluate their association with effector Th subset composition, and correlate Treg subpopulation profiles with objective parameters of immune inflammation, gut permeability, cell metabolism, autonomic nervous system function and HPA axis activity. The findings of this study are intended to identify specific immune and metabolic parameters with the potential to differentiate between ME/CFS and LC.

## 2. Results

### 2.1. Demographic Characteristics of the Study Groups

The demographic characteristics of the study groups are given in Table 1. Overall, 85 males and 209 females were included. The sex distribution did not differ significantly between the study groups (χ^2^ = 1.32, df = 2, *p* = 0.52). Although not reaching statistical significance in this sample, a higher proportion of females was observed in the ME/CFS group, consistent with the known female predominance of this condition reported in epidemiological studies. Age and BMI differed significantly between healthy subjects and the other two groups (*p* < 0.001 and *p* < 0.05, respectively) (Table 1). Therefore, both variables were included as covariates in subsequent adjusted analyses of immune markers.

Participants with long COVID had a shorter median disease duration (23 months) compared to those with ME/CFS (36 months, *p* = 0.001), with sex-specific medians of 25 and 20 months in men and women with long COVID, and 24 and 36 months in men and women with ME/CFS, respectively. Few participants with co-morbidities were included in the study: one in the LC group with recurrent *Herpes zoster* infection, and 7 in the ME/CFS group, as follows: 2 with reactivated *Epstein–Barr* virus infection, 1 with cerebrovascular disease, 1 with cardiovascuar disease, 1 with dysthymia, and 1 with reactive arthritis. Two donors with LC reported antihypertensive treatment and two ME/CFS donors were on antiacid medication during enrollment. Practically all LC and ME/CFS participants reported having taken some supplements during a certain period after the symptoms onset or the positive COVID-19 test (most often vitamin C, A, E, D or combined forms, L-lysine, glutathion, melatonine). Significantly lower shares of LC and ME/CFS participants were vaccinated for COVID-19 compared with HC, most with at least two doses. All LC participants reported mild to moderate COVID-19 symptoms, and no one had been hospitalized. Regarding the period of primary infection, 24% were infected at the end of 2020–beginning of 2021 (alpha variant); 18.8% between July 2021 and December 2021 (delta variant); 58% after December 2021 (Omicron).

### 2.2. Characterization of the T-Cell Pool in LC, and ME/CFS

This stage was intended to identify candidate markers showing overall group differences. The phenotypically defined differentiation status of peripheral blood CD4 and CD8 T cells reflects the presence of acute/chronic immune inflammation as well as the regulatory potential of the immune system. Initial immune-marker screening was performed using one-way ANOVA to compare immunological variables across the three groups.

**Figure 1:**
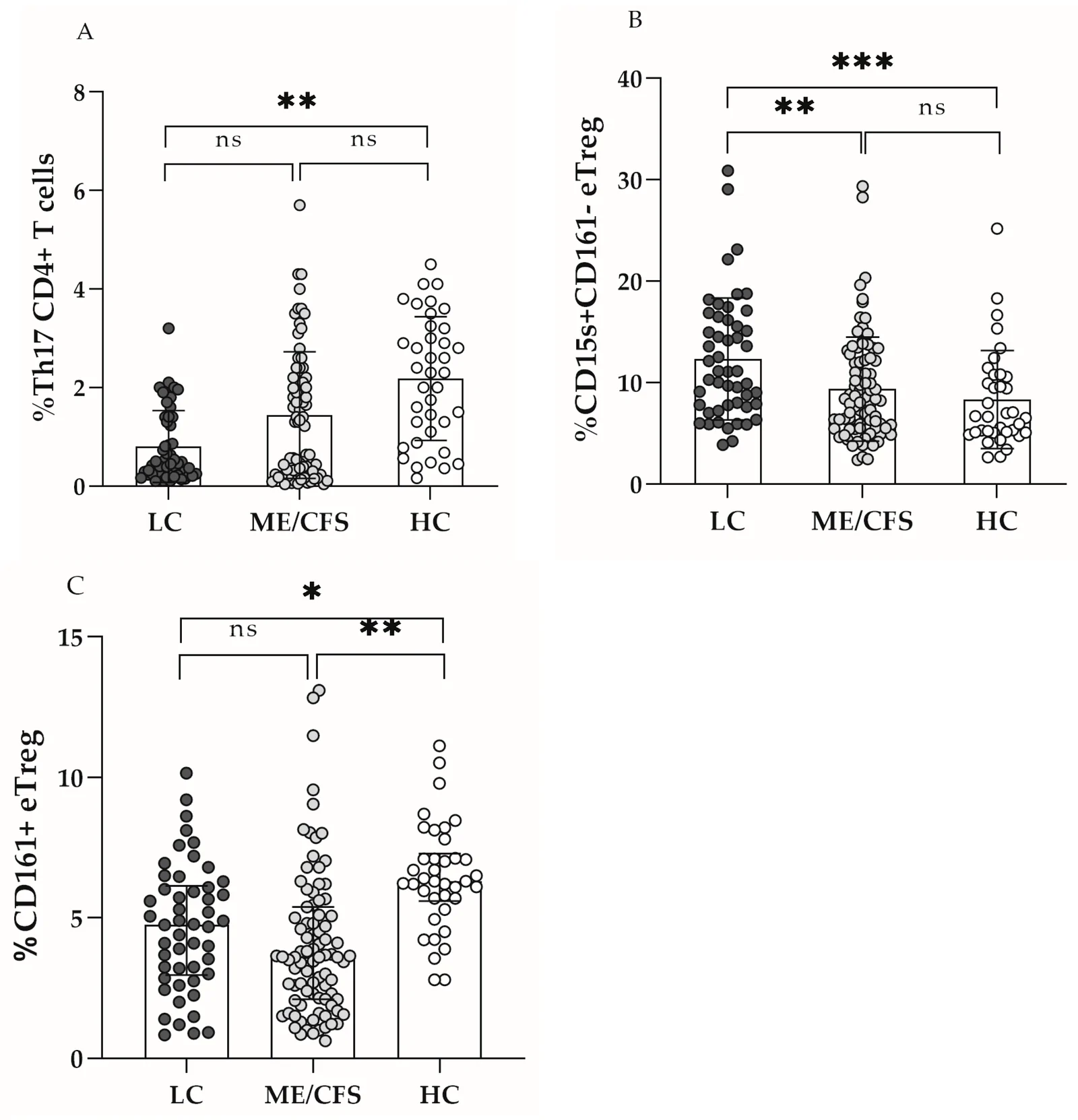
T-cell subsets differentiating between LC (*n* = 50), ME/CFS (*n* = 87) and HC (*n* = 38): scatter plots of Th17 (**A**), CD15s+CD161− Treg (**B**), and CD161+ eTreg (**C**). Bar chart and error bars representing median and interquartile range. Statistically significant differences were determined by the Kruskal–Wallis test, and Dunn–Bonferroni adjustment was applied (ns, not significant, * *p* < 0.05; ** *p* < 0.01; *** *p* < 0.001).

We determined the shares of naïve (N), central memory (CM), effector memory (EM), terminal effector (TE), CD95-expressing and activated (HLA-DR+/CD38+) CD4 and CD8 T in both syndrome groups in comparison to HC. In addition, the effector (Th1, Th2, Th17, Th22, Th9, ThG, Tfh), regulatory (Treg) and transitory (Th1-, Th2-, Th17-, Th22-like) subsets were evaluated. Initial screening (one-way ANOVA or Kruskal–Wallis) identified multiple immune markers that differed significantly among the three groups. The most prominent candidates included Tfh1, Th1, Th17, CD38-HLA-DR+, CD38+HLA-DR+, CD38+HLA-DR-, CD38-HLA-DR-, CD73+nTreg, CD31+eTreg, CD38+eTreg, CD73+eTreg, CD147+eTreg, CD1611+eTreg, GARP+eTreg, PI16+eTreg, CD15s+CD161-eTreg, Th1-like, Tfr, CD4+N, CD4+EM, CD4+CD95+, and CD8+N. After Benjamini–Hochberg FDR correction, the following parameters remained significant: Th17, CD38-HLA-DR+, CD38+HLA-DR-, CD31+eTreg, CD161+eTreg, GARP+eTreg, CD15s+CD161-eTreg, and CD4+CD95+ (Supplementary Table S1). Because age, BMI and sex were potential confounders of immune-cell phenotypes, they were included as covariates in Quade’s ANCOVA for the FDR-prioritized immune markers, with diagnostic groups as the fixed factor. After adjustment, few biomarkers remained significantly associated with diagnostic groups (Table 2). The Th17 subset was decreased in both syndrome groups, reaching statistical significance only for LC compared to HC, median [Q1–Q3]: 0.73 [0.21–1.41] vs. 2.40 [1.15–3.55], *p* = 0.003. LC donors displayed a significantly increased CD15s+CD161-Treg subset: 14.74 [10.27–18.73] vs. 9.87 [6.28–14.97] for ME/CFS and 5.25 [4.28–9.10] for HC (*p* = 0.005; and *p* = 0.010, respectively). In contrast, CD161+eTreg decreased in both LC (5.45 [3.54–6.47]) and ME/CFS (4.50 [2.20–6.50]) compared to HC (6.30 [5.78–7.28]) (*p* = 0.029 and *p* = 0.004, respectively) (Figure 1A–C, Table 2, Supplementary Table S1).

Thus, in both pathologic conditions, LC in particular, a decrease in Th effectors producing inflammatory Th17 cytokines was observed, alongside an excess of Treg with a phenotype attributed to strong and stable suppressors. These changes were at the expense of the CD161+Treg subset characterized by plasticity and proinflammatory activity [22,23].

### 2.3. T-Cell Subsets, Chronic Activation and Immune Cell Metabolism

**Figure 2:**
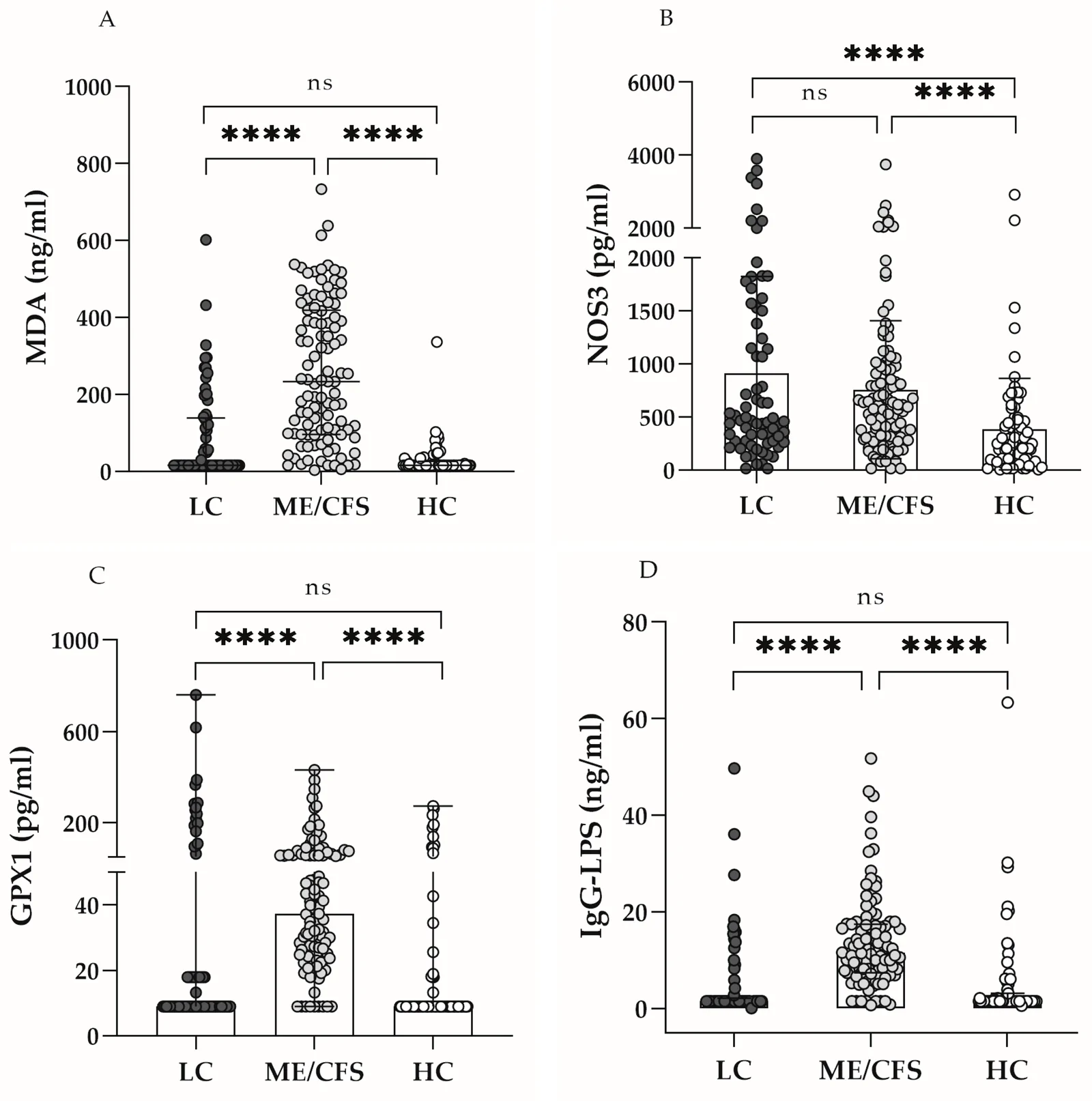
Serum levels of MDA (**A**), NOS3 (**B**), GPX1 (**C**) and IgG-LPS (**D**) in participants with LC (*n* = 69), ME/CFS (*n* = 104) and HC (*n* = 75) shown in scatter plots with bar charts and errors bars representing the median and interquartile range. Statistically significant differences were determined by the Kruskal–Wallis test, and Dunn–Bonferroni adjustment was applied (ns, not significant, **** *p* < 0.0001).

To further study the state of chronic immune activation and related metabolic changes, we evaluated established soluble markers of increased intestinal permeability and activation (LPS, IgG-LPS, zonulin, TLR4); redox and repair potential (GPX1, SOD1, CAT, DKK1); and oxidative and nitrosative stress (MDA, NOS1, NOS3). While most studied serum markers were significantly altered in patient groups (Supplementary Table S1), only a few survived age/BMI/sex adjustments (Table 2). MDA (median [Q1–Q3]: 233.60 [96.23–419.08]) and GPX1 (38.15 [24.38–59.71]) were significantly increased in ME/CFS compared to both LC (15.62 [15.62–141.60], 8.97 [8.97–29.54]) and HC (15.62 [15.62–26.22], 8.97 [8.97–13.25]), while high levels of NOS3 (478.55 [272.10–1506.75]) distinguished LC donors from the other two groups (604.75 [306.20–978.85], 255.70 [101.20–473.80]) (Supplementary Table S1). MDA, GPX1 and NOS3 remained significantly associated with diagnostic groups after adjustment for age and BMI, while IgG-LPS showed significant age and BMI associations (Table 2, Figure 2).

To further address the potential influence of age, BMI, and sex on the findings, ANCOVA surviving biomarkers were reanalyzed using overlap-restricted samples. Only participants aged 30 to 65 years (Table 3), with a BMI of 18 to 30 (Table 4), or of similar gender were included in these analyses (Supplementary Figures S1 and S2). MDA, GPX1, Th17, CD161+Treg and CD15s+CD161−eTreg remained significantly different between the age- and BMI-restricted groups. In contrast, NOS3 was age-dependent, while CD161+eTreg differentiated only between ME/CFS and HC after BMI restriction. All biomarkers remained significantly different between the study groups in the male-only and female-only cohorts.

**Figure 3:**
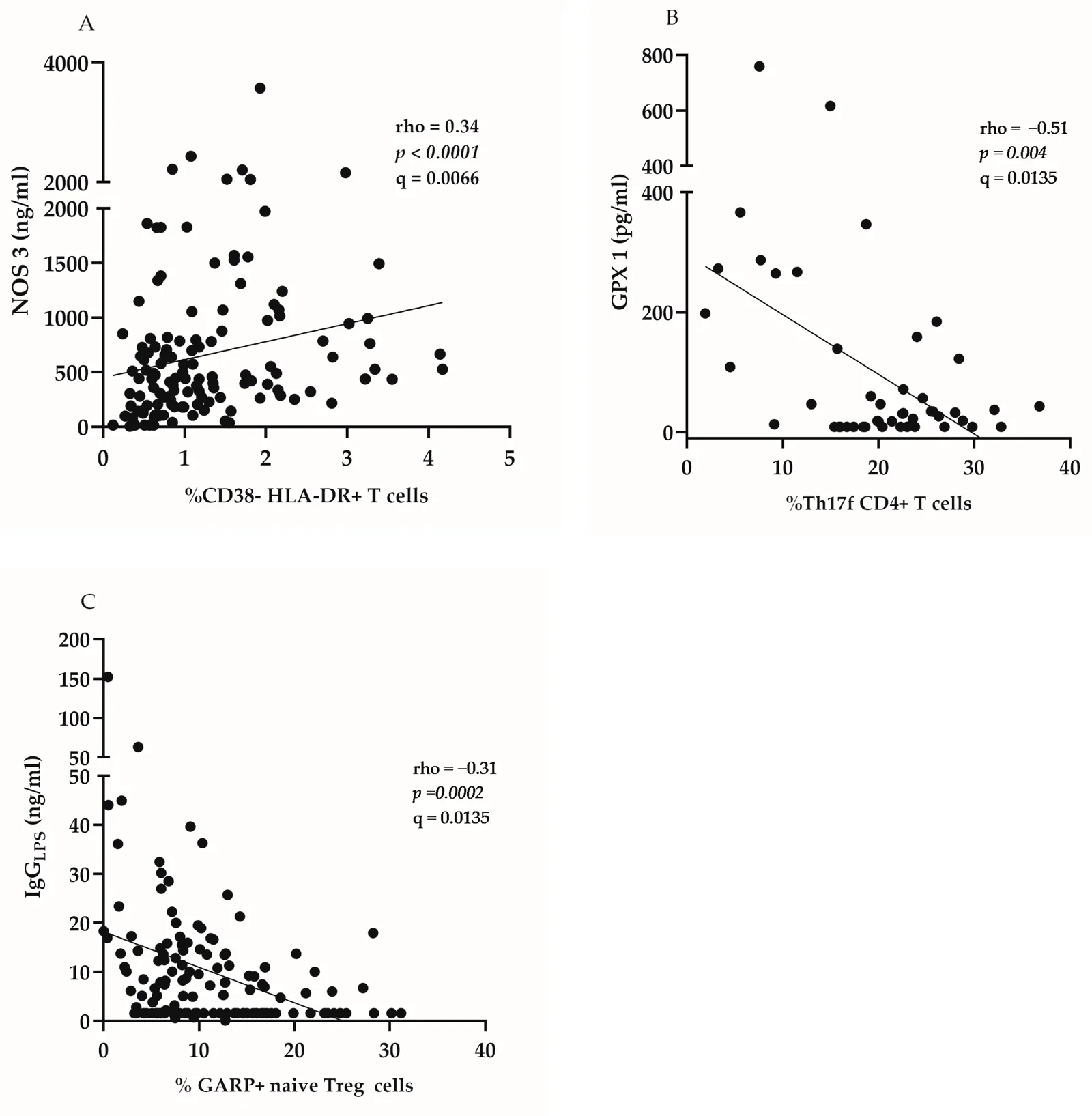
Correlations between T-cell subsets and soluble markers of cellular metabolism and chronic activation: NOS3 (**A**), GPX1 (**B**) and IgG-LPS (**C**). Correlation coefficients were calculated using Spearman’s method. Significance levels were adjusted for multiple comparisons using the FDR correction.

We further looked for specific associations between the studied T-cell subsets and soluble markers. In the whole cohort, a direct significant association was observed between CD38−HLA-DR+ T and NOS3 (r = 0.340, *p* < 0.001). Tfh17 correlated strongly and inversely with GPX1 (rho = −0.504; *p* < 0.001), and GARP+ nTreg showed significant inverse associations with IgG-LPS (rho = −0.312, *p* < 0.001) and GPX1 (rho = −0.271, *p* < 0.01), (Figure 3A–C). Since Th17 T and GARP+e Treg did not remain independently significant after age/BMI adjustment, the latter findings were interpreted as exploratory.

### 2.4. T-Cell Subsets and Clinical Symptoms Measures

Next, we looked for a possible association between altered T-cell subsets and clinical symptom severity. PS-HAMA, T-FF, Pu-FF, DePol-Tot, and T-HAMA differed significantly among the LC, ME/CFS, and HC groups (one-way ANOVA and FDR correction): PS-HdAMA, DePol-Tot and T-HAMA were higher in both disease groups compared with HC, without a significant difference between LC and ME/CFS. In contrast, T-FF and Pu-FF showed a graded pattern, with LC having the highest scores, ME/CFS having intermediate scores, and HC having the lowest scores. Spearman correlations were calculated between selected immune biomarkers (differentiating between groups) and the five FDR-significant mental health variables. The analysis was performed in the whole cohort and in the disease cohort only, including LC and ME/CFS subjects and excluding HC. Benjamini–Hochberg FDR correction was applied separately in each cohort across 30 correlations. No immune–mental health correlation survived FDR correction at q < 0.05 (Table 5).

### 2.5. Classification Analyses of Identified T-Cell and Soluble Biomarkers

The five biomarkers surviving the age- and BMI-restricted comparison—Th17, CD15s+CD161−eTreg, CD161+eTreg, MDA and GPX1—were further analyzed for their classification performance. An exploratory three-class Random Forest with nested cross-validation performance and multiple imputation by chained equations (MICE) inside every fold model showed moderate global classification performance of the selected markers, with a mean accuracy of 0.595, a balanced accuracy of 0.576, an F1 (95% CI) of 0.567 (0.514–0.614), and an AUC (95% CI) of 0.812 (0.778–0.843) (Supplementary Table S2). The following degree of missingness was present: Th17, CD161+eTreg, and CD15s+CD161−eTreg in 119 participants (40.5%), and MDA and GPX1 in 45 other participants each (15.3%). Among 80 HC participants, 56 were classified correctly as HC, 10 as LC, and 14 as ME/CFS. Among 89 LC participants, only 30 were classified correctly, whereas 34 were classified as HC and 25 as ME/CFS. Among 125 ME/CFS participants, 92 were correctly classified, with 13 classified as HC and 20 as LC. In the three-class model, GPX1 and MDA were the two dominant features, with permutation importance values ± SD of 0.121 ± 0.038 and 0.112 ± 0.025, respectively. CD15s+CD161−eTreg ranked third (0.023 ± 0.012), followed by CD161+eTreg (0.012 ± 0.018) and Th17 (0.010 ± 0.014) (Table 6, Supplementary Table S3).

In pairwise Random Forest models for LC vs. HC, ME/CFS vs. HC, and ME/CFS vs. LC, the strongest discrimination was observed between ME/CFS and HC. In 205 participants, the repeated cross-validation balanced accuracy was 0.802, with an overall accuracy of 0.809 and an AUC (95% CI) of 0.907 (0.865–0.944). For ME/CFS versus HC, MDA was clearly dominant, with a permutation importance ± SD of 0.210 ± 0.033, markedly exceeding GPX1 (0.059 ± 0.020), CD161+eTreg (0.008 ± 0.006), Th17 (0.002 ± 0.006) and CD15s+CD161−eTreg (0.000 ± 0.005) (Table 6, Supplementary Tables S2 and S3).

Discrimination between ME/CFS and LC was also substantial, although weaker than that between ME/CFS and HC. Among 214 participants, the balanced accuracy was 0.759, overall accuracy 0.761, and AUC (95% CI) 0.862 (0.814–0.904). Conversely, GPX1 became the leading discriminator of ME/CFS versus LC, with an importance ± SD of 0.188 ± 0.037, followed by MDA (0.080 ± 0.023), CD15s+CD161-eTreg (0.015 ± 0.012), Th17 (0.003 ± 0.006) and CD161+eTreg (0.000 ± 0.004) (Table 6, Supplementary Tables S2 and S3).

The most difficult binary comparison was LC versus HC. Among 169 participants, the balanced accuracy was 0.664, accuracy 0.662, and AUC (95% CI) 0.727 (0.658–0.791). For LC versus HC, MDA had the highest permutation importance ± SD (0.095 ± 0.043), followed by CD15s+CD161−eTreg (0.037 ± 0.032), Th17 (0.033 ± 0.023), GPX1 (0.024 ± 0.021), and CD161+eTreg (0.006 ± 0.017) (Table 6, Supplementary Tables S2 and S3).

Based on the above results, the chosen biomarker set was informative regarding group membership, but the classification produced was not accurate enough to be interpreted as a stand-alone diagnostic classifier.

Keeping in mind the high degree of data missingness and the artificial structure of imputed data, we preferred to avoid logistic regression analysis of the whole data set. Instead, a binary logistic regression was performed with the two most complete data sets, MDA and GPX1, without any imputations. The ME/CFS vs. HC model was the strongest logistic regression model (χ^2^ (2) = 123.38, *p* <0.0001), with excellent discrimination. The model explained approximately 67.0% of the variance (Nagelkerke R^2^ = 0.670) and correctly classified 92.4% of cases. Both MDA (OR = 8.300, 95% CI [4.417–15.597]) and GPX1 (OR = 1.719, 95% CI [1.089–2.713]) were independent predictors. The ME/CFS vs. LC model was significant but weaker than the ME/CFS vs. HC model (χ^2^ (2) = 50.82, *p* < 0.001) and correctly classified 75.9% of cases. MDA was the only significant independent predictor (OR = 3.074, 95% CI [2.063–4.581]), whereas GPX1 (OR = 1.304, 95% CI [0.900–1.890]) was not significant. Finally, the LC vs. HC model was significant (χ^2^ (2) = 12.65, *p* = 0.002) but weaker than the other two models. The model explained approximately 11.1% of the variance (Nagelkerke R^2^ = 0.111) and correctly classified 57.7% of cases. MDA (OR = 1.831, 95% CI [1.163–2.883]) was an independent predictor, whereas GPX1 (OR = 1.070, 95% CI [0.702–1.629]) was not significant. Thus, logistic regression identified MDA as the most consistent independent predictor across all pairwise comparisons. In addition, the high accuracy of the ME/CFS vs. HC comparison (AUC 0.924) confirmed that the Random Forest result AUC = 0.907 was biologically consistent, and not an imputation artifact (Table 7, Supplementary Table S4).

## 3. Discussion

In this cross-sectional study, we hypothesized that the principal pathological mechanism underlying LC and ME/CFS is a failure of immune regulation associated with ongoing immune activation, leading to profound changes in immune cell metabolism. By performing a detailed analysis of T-cell subsets and soluble biomarkers, we identified a biologically coherent immune-serum signature distinguishing LC, ME/CFS, and HC, comprising Th17, CD15s+CD161−eTreg, CD161+eTreg, MDA, and GPX1. The present findings are consistent with an expanding body of evidence demonstrating that ME/CFS and long COVID are multisystem post-infectious disorders involving immune, redox, vascular, autonomic, and metabolic abnormalities rather than purely psychiatric or functional syndromes [15,30]. A study directly comparing lymphocyte subsets and cytokine profiles in 190 participants found that ME/CFS and long COVID display comparable immune and inflammatory profiles, with no significant differences between the two disease groups in most tested biomarkers [31]. More recently, a systematic transcriptomic analysis showed that shared molecular pathways—particularly those involving immune regulation, mitochondrial function, and energy metabolism—underlie both conditions [32].

Both ME/CFS and LC are multifaceted conditions. A number of factors add to the inherent heterogeneity of these pathologies, possibly leading to different and even contradictory immune profiles. Age and BMI are major confounders, as they are naturally associated with chronic inflammation and immune activation, supposedly driving the pathogenesis of CFS and LC. The female preponderance in both ME/CFS and LC suggests that biological sex significantly influences disease susceptibility, symptom severity, and underlying pathophysiology [33].

In our study, age represented a substantial potential confounder because healthy controls were significantly younger than both disease groups. Sensitivity analyses confirmed that several markers did not remain independently associated with the diagnostic group after age/BMI/sex adjustment. This was especially evident for CD38+/HLA-DR+ T subsets, NOS3, CD31+eTreg, and GARP+eTreg, which should therefore be considered secondary age-sensitive immune features rather than core diagnostic biomarkers.

In contrast, the five-marker signature (MDA, GPX1, Th17, CD15s+CD161−eTreg, and CD161+eTreg) remained significant after demographic adjustment.

Although the cohort showed marked female predominance, sex distribution did not differ significantly across diagnostic groups, and all five primary biomarkers remained significantly associated with the diagnostic group after sex adjustment. Our results indicate that the main diagnostic-group findings were not driven by sex composition. However, it should be acknowledged that the very small number of male participants can mask significant between-group differences, and therefore, this conclusion requires confirmation in a more balanced cohort.

In addition, a number of factors were shown to shape the post-COVID state, including the number and severity of SARS-CoV-2 infections, pandemic wave, and vaccination status [2,33]. We have previously shown that long-term T-cell memory to SARS-CoV-2 after natural and combined exposure has different characteristics [34], and may therefore affect the pathophysiology of LC. Although a small number of LC donors reported COVID vaccination, they could have attributed to the heterogeneity of the studied group and may have influenced immune phenotypes and related serum biomarkers. This fact is acknowledged as a limitation of the study.

The multitude of cellular and serum markers analyzed in this study cover several consequently related biological domains: pathogen- and microbial-translocation-related immune reactivity, the resulting T-cell activation, regulatory T-cell alterations, Th17/Treg axis disbalance, and systemic oxidative stress. Notably, the finally selected panel was associated with the last three, indicating that LC and ME/CFS result from continuous and lasting changes.

Th17 and Tregs are functionally opposed CD4+ T-cell lineages whose reciprocal differentiation from naïve precursors is governed by the competing transcription factors RORγt and Foxp3. Th17/Treg imbalance disrupts immune tolerance, promotes mucosal inflammation, and amplifies responses to gut-derived microbial signals [35]. In the present study, Th17 displayed one of the largest ANCOVA age- and BMI-adjusted group effects of all five core biomarkers (partial η^2^ = 0.253 and 0.241), with a significant decrease in LC compared to ME/CFS and HC. Flow-cytometric analyses in ME/CFS have previously documented an increase in the Th17 population and reduced cytokine production in other T-cell subsets, features interpreted as signatures of chronic immune exhaustion [36]. A study simultaneously profiling ME/CFS and LC using transcriptomics found that Treg abundance was significantly lower in ME/CFS than in LC, while exhausted CD8 T-cell profiles showed the opposite pattern [37], highlighting the complementarity of Th17/Treg profiling for disease differentiation.

Long COVID was associated with a Th2/Th17 bias, consistent with a loss of immune tolerance that may be driven by molecular mimicry or persistent viral antigens [38]. Decreased Treg numbers and impaired Treg function have also been linked to Treg/Th17 imbalance and respiratory failure in acute severe COVID-19 [39]. Notably, in line with our results, Wiech et al. demonstrated a significant decrease in IL-17 alongside Th1 cytokines, persisting in moderate COVID-19 convalescents at 3 and 6 months post-infection. This was accompanied by an increase in naïve Tregs, suggesting that the Th17/Treg dysregulation observed chronically in ME/CFS and LC cohorts may represent a maintained post-infectious immune state [40]. Th17 cells play an important role in mucosal immunity, epithelial barrier maintenance, and protection against extracellular pathogens [41]. While several studies of acute COVID-19 have demonstrated elevated Th17-associated cytokines during severe infection [42,43], the reduced Th17 frequencies observed in the present LC cohort may indicate a transition from acute inflammatory activation toward a state of chronic immune exhaustion or compensatory downregulation. Persistent inflammatory stimulation may ultimately impair Th17 maintenance and differentiation, resulting in reduced adaptive flexibility and impaired tissue immune surveillance.

A major finding of the present study is that total Treg frequencies did not differ significantly between LC, ME/CFS, and HC, whereas substantial alterations were detected within Treg subsets. This observation supports the concept that qualitative rather than quantitative Treg abnormalities may drive chronic immune dysregulation [16].

The most pronounced between-group differences were observed for CD15s+CD161−eTreg, CD161+eTreg, GARP+eTreg and CD31+eTreg. Importantly, the changes in the first two subsets were age/BMI independent. The CD15s+CD161−eTreg phenotype is of particular interest, as it has been shown to define the most highly differentiated, stable, and potently suppressive subpopulation of effector human Tregs [22,23]. On the other hand, CD161 (NKR-P1) expression defines a Th17-biased progenitor “plastic” state in Tregs [23]. The significantly increased CD15s+CD161−eTreg in both pathologies, particularly in LC, at the expense of CD161+eTreg, suggests an effort to counteract important and continuous activation signals that might affect plasticity and the capacity for prompt protective immune response to new stimuli. Notably, CD15s+CD161− and Th17 phenotypes contributed most significantly to LC vs. HC discrimination, suggesting that Treg and Th17 subsets are significant biomarkers.

Although the IgG-LPS elevation observed in ME/CFS donors did not survive age and BMI adjustment, it should be kept in mind as an essential driving factor for immune inflammation. IgG-LPS is an established indirect marker of gut-barrier dysfunction and microbial translocation. IgA responses against the LPS of commensal enterobacteria in ME/CFS patients have been shown to correlate with illness severity [44] and to predict long COVID status [45]. A more recent multicenter cross-sectional study measuring LPS alongside anti-beta-lactoglobulin antibodies, zonulin-1, and soluble CD14 in 22 fibromyalgia (FM), 30 ME/CFS, and 26 HC participants found significantly elevated levels of all gut-barrier biomarkers in ME/CFS relative to HC, with strong ROC curve discrimination (LPS AUC = 0.806 [46].

Correspondingly, in the present study, we identified significant associations between GARP+nTreg and IgG-LPS (rho = −0.312, q = 0.0135) on one hand, and between GARP+nTreg and TLR4 (rho = 0.276, q = 0.0416) on the other, suggesting that microbial-translocation-related signaling modulates Treg phenotype.

CD38 and HLA-DR co-expression on T cells has long served as a phenotypic hallmark of late-stage T-cell activation and is characteristic of antigen-driven immune responses. In the present study, the CD38−HLA-DR+ disease-associated activation phenotype was rather superimposed on an age-related immune-activation background. Importantly, however, this activation phenotype correlated with NOS3 (Spearman’s rho = 0.340, q = 0.007), linking T-cell activation to the endothelial-redox pathway, consistent with the known capacity of IFN-γ produced by activated T cells to upregulate endothelial NOS3 expression while simultaneously promoting its uncoupled, superoxide-generating state [47].

The CD38+HLA-DR+ double-positive phenotype has been identified as a marker of hyper-immune activation in severe COVID-19 [48] and is independently associated with long COVID symptom burden [49]. Phetsouphanh et al. found elevated levels of activated innate immune cells and T-cell perturbations for up to eight months after initial mild-to-moderate SARS-CoV-2 infection [50]. These data corroborate the present finding that elevated CD38−HLA-DR+ (more importantly in LC than in ME/CFS) characterizes the two syndrome groups relative to HC.

Interestingly, CD38 expression was lowest in LC, suggesting a potentially atypical activation profile associated with prolonged immune stimulation and dysfunctional immune adaptation syndrome. Persistent antigen exposure may induce atypical activation profiles, as already demonstrated after viral infections [26,27]. We propose that the dissociation between elevated HLA-DR expression and reduced CD38 levels may reflect possible exhaustion-associated dysregulation and progressive functional dysregulation.

An important aspect of the present study is the identification of associations between altered T-cell subsets and oxidative/nitrosative stress pathways. Oxidative stress has been increasingly recognized as a key contributor to chronic inflammation and post-viral symptom persistence [51]. The inverse immune–serum correlation between Tfh17 and GPX1 (rho = −0.504, q = 0.0135) is mechanistically consistent with the known antagonistic relationship between Th17 activity and antioxidant defense: IL-17-mediated ROS production depletes glutathione peroxidase substrates, while GPX1-mediated clearance of hydrogen peroxide reduces Th17 differentiation signals. A mechanistic hypothesis consistent with the findings integrates the Th17 and MDA axes into a single pathophysiological circuit in which Th17 expansion depletes antioxidant capacity, thereby permitting further lipid peroxidation and MDA accumulation, which in turn sustains oxidative-stress-driven immune activation. The increased CD38-HLA-DR+ T-cell subset correlated significantly with NOS3, suggesting that ongoing immune activation observed in LC is related nitrosative to stress. Nitrosative stress has previously been implicated in mitochondrial dysfunction, autonomic dysregulation, and neurocognitive symptoms in ME/CFS [52]. In addition, the inverse correlation of GARP+nTreg with IgG-LPS links the changes in phenotypic regulatory subsets to microbial LPS-specific immune response and mechanistically supports the above-discussed concept of lost immune regulation capacity in response to microbial translocation and continuous immune activation.

In our study, MDA, GPX1, and NOS3 were the best differentiating serum biomarkers, with significant elevations in symptom groups. ME/CFS exceeded LC in MDA and GPX1 expression, while NOS3 was the most elevated in LC. Importantly, the elevated expression of MDA and GPX1 in ME/CFS and of NOS3 in LC proved independent of age, BMI and sex in ANCOVA analysis, while age-restricted analysis showed that NOS3 is age-sensitive. MDA, the principal end-product of lipid peroxidation and a biomarker of oxidative damage, was repeatedly found elevated in ME/CFS relative to HC, implicating dysregulation of lipid metabolism, mitochondrial function, and antioxidant defenses [53,54,55]. Significant increases in MDA described in ME/CFS patients were associated with alterations in erythrocyte morphology, consistent with oxidative membrane damage [56]. In the context of LC, MDA and related lipid-peroxidation markers were shown to persist in subjects with previous COVID-19 infection relative to asymptomatic post-COVID controls, and a Random Forest classifier using MDA serum levels achieved the best separation of long-COVID from asymptomatic post-COVID subjects [57]. This finding closely parallels the present study, in which MDA ranked first in permutation importance for the ME/CFS versus HC and LC vs. HC pairwise comparisons, and second in the three-class classification, together with GPX1 (permutation importance values 0112 vs. 0.121).

GPX is a Se-dependent enzyme that reduces hydroperoxides by oxidizing glutathione and consequently protects cells from oxidative damage. In patients with LC, GPX1 deficiency was associated with the accumulation of free radicals, lipid peroxidation and protein damage. It was shown that the impact of acute COVID-19 on the symptoms of long COVID is partly mediated by impaired antioxidant mechanisms, including lowered GPX [58]. Certain genetic variants of GPX1, independently or in combination with others, have been shown to affect the probability of long-COVID manifestations, particularly “brain fog” [59]. In a study by Maes et al., GPX activity was significantly lower in depressed patients than in normal controls, while in the context of ME/CFS, no significant differences were found [60]. Interestingly, according to our results, GPX1 levels in LC were comparable to HC, while the ME/CFS group was distinguished by significantly elevated GPX1 levels independent of any demographic parameters, in parallel with elevated MDA. This apparent paradox reveals that the association between GPX1plasma level, GPX1 activity, and oxidative stress damage is not so straightforward. A plausible explanation may be that, in the long run, 1. the availability of glutathione decreases, while GPX1 needs glutathione in order to function, or 2. in spite of high serum concentration, the enzyme is not active/functional. Thus, the simultaneous accumulation of GPX1 and MDA may characterize an advanced stage of cellular metabolic dysregulation in ME/CFS.

This concept is confirmed by the excellent discriminatory power of the binary MDA/GPX1 logistic model for ME/CFS vs. HC. Both MDA (OR = 8.300, 95% CI [4.417–15.597]) and GPX1 (OR = 1.719, 95% CI [1.089–2.713]) were independent predictors.

MDA also independently separated ME/CFS from LC (OR = 3.074, 95% CI [2.063–4.581]), raising the possibility that the oxidative stress burden is more pronounced or more firmly established in ME/CFS than in the LC cohort. On the other hand, LC vs. HC separation involved, in addition to MDA, two T-cell subsets: CD15sCD161-Treg and Th17, indicating a different (earlier?) level of dysregulation, which may still be compensated through corrections of Treg/Th17 balance. Based on these results, it is tempting to speculate that the observed differences represent temporal progression, consistent with the longer average disease duration in ME/CFS patients in the present sample. Previous research has already shown that immune changes in ME/CFS vary significantly based on disease duration, from a highly active hyper-inflammatory state to a state of immune exhaustion or depletion over time [61,62]. Although T-cell biomarkers seemed considerably less powerful as predictors, this could also be a consequence of the high level of data missingness affecting these two subsets in particular. Therefore, external validation with adequate sample sizes and matched disease durations is required to determine whether MDA reliably differentiates ME/CFS from LC beyond the present cohort.

Increasing evidence links persistent immune activation and inflammatory cytokine signaling to depression and anxiety following viral infection [60,63]. Pro-inflammatory mediators can influence neurotransmitter metabolism, microglial activation, hypothalamic–pituitary–adrenal axis regulation, and neural network function, thereby contributing to mood disturbances and cognitive dysfunction [52]. In this context, altered Treg functionality may play a central role by failing to adequately suppress persistent inflammatory and oxidative pathways post-infection.

The mental health analyses identified a focused set of five FDR-significant variables: PS-HAMA (somatic anxiety), T-HAMA (total anxiety), DePol-Tot (total depolarization score), T-FF (total FibroFatigue score), and Pu-FF (pure FibroFatigue). These variables were strongly intercorrelated. Notably, T-FF and Pu-FF showed a graded LC > ME/CFS > HC pattern, whereas PS-HAMA, T-HAMA, and DePol-Tot were elevated in both disease groups relative to HC, suggesting that fatigue symptoms are more specific than general anxiety or autonomic perturbation measures.

A critical finding of the present study is that no immune–mental health correlation survived FDR correction in either the whole cohort or the disease-only (LC + ME/CFS) analysis, despite important group differences in both domains. Our results are inconsistent with a simple model in which immune biomarkers directly drive or proportionally reflect symptom severity. Elevated MDA or altered Th17/Treg ratios cannot explain the observed anxiety and fatigue burden through direct neuro-immune pathways. The fact that Maes et al. demonstrated that oxidative indices in LC post-viral syndrome were associated with the physio-affective phenomena (fatigue, depression, anxiety) [58] suggests that the relationship depends on biomarker selection, population, and analytical stringency.

Published data does not uniformly predict strong immune–symptom correlations in ME/CFS or long COVID. A systematic review by Wortinger et al. in ME/CFS found that whereas cytokines (IL-1, IL-2, IL-6, TNF-α) showed some correlations with executive function, emotion regulation, and sleep quality, findings were inconsistent across studies, effect sizes were modest, and there was an almost complete absence of longitudinal data [63]. Crucially, the present study measured flow cytometry-derived T-cell phenotypes rather than soluble cytokines—which may have a more distal relationship with moment-to-moment symptom experience than circulating inflammatory mediators. The moderate nominal correlation between MDA and DePol-Tot (rho = 0.218, raw *p* = 0.029) in the whole cohort, which did not survive FDR correction and disappeared in the disease-only cohort, may have been driven partly by disease-control contrast rather than genuine within-patient symptom coupling. In fact, the symptom-burden cluster may be more directly driven by neural, autonomic, metabolic, or microstructural brain alterations than by peripheral immune biomarkers, consistent with emerging evidence for neuroinflammatory and neurometabolic correlates of fatigue and cognitive impairment in ME/CFS and long COVID [1,64,65]. Also, individual variability in symptom reporting scales, illness adaptation, and comorbid psychological conditions may introduce noise into cross-sectional immune–symptom associations that would only resolve with longitudinal repeated-measures designs.

The present finding therefore supports a two-domain model: the immune-serum markers define a biological disease phenotype, while the mental health and symptom burden variables define a clinical experience phenotype. These domains are likely linked through complex, indirect, and potentially non-linear pathways that cross-sectional correlation analyses in heterogeneous mixed cohorts may lack the power to detect.

A central question in post-infectious syndrome research is whether ME/CFS and LC are immunologically distinct entities, overlapping post-viral phenotypes, or endotypes within a broader spectrum of post-acute infection syndromes. According to the present classification analyses, ME/CFS showed strong, consistent separation from HC across Random Forest (AUC = 0.907) and logistic regression (AUC = 0.924), whereas LC showed substantially more modest separation from HC (AUC = 0.727 and 0.577 respectively), and the pairwise ME/CFS versus LC comparison yielded intermediate discrimination (Random Forest AUC = 0.862; logistic AUC = 0.777). In the three-class confusion matrix, only 57% of LC cases were correctly classified. The possible interpretation is that LC is a biologically heterogeneous syndrome; the pathophysiological mechanisms driving LC symptomatology may vary across patients and may not uniformly engage the oxidative-stress and Th17/Treg pathways targeted by the present panel; or additional factors (time since infection, SARS-CoV-2 viral load, vaccination history) may dilute the biomarker signal in LC [33]. In line with our findings, Dhingra et al. identified three inflammatory LC clusters: a limited immune activation cluster, an innate immune activation cluster, and a systemic immune activation cluster, with no single inflammatory profile characterizing all LC patients [66] Similarly, three clinically distinct LC phenotypes were described at 3–6 months post-infection [67]. A systematic review of LC studies identified considerable endotypic diversity, reflecting the heterogeneous recruitment strategies, timepoints, and outcome measures employed [68].

By contrast, ME/CFS showed greater immunological homogeneity in the present data, possibly reflecting the longer chronicity of the disease or a restricted set of triggering infectious events. This hypothesis is supported by data that ME/CFS immune abnormalities—including NK cell dysfunction, cytokine alterations, and T-cell changes—show moderate reproducibility across independent cohorts, whereas LC immune profiles show greater inter-study heterogeneity [32]. The convergence of MDA elevation across both diseases, with greater magnitude in ME/CFS, evokes the hypothesis that oxidative stress may represent a shared pathological mechanism, though more advanced in ME/CFS. Since the cross-sectional design of the study does not allow for determining whether the observed differences represent temporal progression, distinct pathophysiological processes, or cohort differences, such a hypothesis should be further tested in longitudinal studies.

Collectively, the present study supports the concept that LC and ME/CFS are characterized by persistent immune remodeling involving both adaptive immune activation and dysfunction of regulatory pathways. LC demonstrated a more pronounced phenotypically altered Treg profile and Treg/Th17 disbalance, potentially reflecting ongoing post-SARS-CoV-2 immune perturbation. On the other hand, ME/CFS was characterized by a parallel elevation of GPX1 and MDA, evoking seriously perturbed metabolic regulation.

Finally, the identified disease-related biomarkers justify future studies on biologically informed personalized therapeutic approaches in ME/CFS and long COVID. Thus, high MDA and GPX1 would justify antioxidant supplementation trials (N-acetylcysteine, CoQ10, glutathione precursors), while targeting CD15s+CD161−eTreg could alleviate suppression and restore the effector T-cell pool plasticity.

Of course, the above-described prediction models are entirely exploratory and require external validation in an independent cohort with balanced age, BMI, and disease-duration distributions. Furthermore, longitudinal studies should determine whether the selected 5 markers—MDA, GPX1, Th17 and CD15sCD161-Treg—are stable disease traits.

## 4. Materials and Methods

### 4.1. Patient Characteristics and Study Design

This study was conducted and reported following STROBE guidelines for observational research. It was a cross-sectional comparative study designed to evaluate T-cell subpopulation differences among three groups: long COVID syndrome, ME/CFS, and healthy controls. The dataset included demographic variables, clinical variables, mental health and symptom-related indicators, serum/soluble biomarkers, and flow cytometry-derived T-cell subpopulation variables. The study included a total of 294 participants, divided into 3 subgroups: LC (*n* = 89), ME/CFS (*n* = 125) and healthy controls (HC) (*n* = 80) (Supplementary Figure S3). Participants were recruited between June 2023 and December 2024 at the National Centre of Infectious and Parasitic Diseases, Sofia, Bulgaria and Medical University Plovdiv, Plovdiv, Bulgaria, after signing informed consent. The study was conducted in accordance with the Declaration of Helsinki and approved by the Scientific Ethics Committee at the Medical University of Plovdiv, Protocol No. 3/30 May 2023, for studies involving humans.

#### 4.1.1. Inclusion Criteria

ME/CFS patients were included in the study according to the diagnostic criteria proposed by the Institute of Medicine (IOM) in 2015, based on the following three symptoms: 1. Significant reduction or limitation in the ability to perform pre-illness levels of daily activities that persists for more than 6 months, accompanied by fatigue that is often profound, of recent or definite (not lifelong) onset, and not substantially relieved by rest. 2. Post-exertional malaise. 3. Unrefreshing sleep. At least one of the following was also required: cognitive impairment or orthostatic intolerance [3,4,5]. LC group participants were categorized based on a positive SARS-CoV-2 PCR test and persistence of symptoms (fatigue/post-exertional malaise, dyspnea, joint pain, cognitive impairment, sleep disturbances, cardiovascular and gastrointestinal symptoms) that cannot be explained by other conditions for more than 3 months after recovery [3,4,5]. Healthy subjects were enrolled based on the absence of an active SARS-CoV-2 infection, confirmed by negative PCR/anti-SARS-CoV-2 IgG, and absence of: LC/CFS symptoms, systemic autoimmune diseases, administration of immunostimulatory or immunosuppressive drugs, history of chronic disease (including diabetes, impaired renal or hepatic function), hospitalization or viral/bacterial infection in the last 2  months preceding the study, abnormal complete blood count and/or biochemical status, increased C-reactive protein, chronic HIV infection, alcohol addiction and other dependencies.

#### 4.1.2. Exclusion Criteria

Pregnant and breastfeeding women and subjects who suffered from psychiatric disorders such as major depressive disorder, generalized anxiety disorder, bipolar disorder, panic disorder, schizo-affective disorder, schizophrenia, psycho-organic syndrome, and substance use disorders (except tobacco use disorder (TUD)) were excluded from the study. We also excluded patients with cancer, systemic autoimmune diseases, neurodegenerative diseases (e.g., multiple sclerosis, Parkinson’s or Alzheimer’s disease) and stroke, as well as those with renal and liver disease. Medication, including antidepressants (SSRIs), low-dose naltrexone, antihistamines, corticosteroids, beta-blockers, and antivirals in the last 2 months preceding the study, was an exclusion criterion for both patient groups.

### 4.2. Sample Collection and Processing

Whole peripheral blood (6 mL) was collected into sodium heparinized collection tubes between 9 a.m. and 12 p.m. Peripheral mononuclear cells (PMNC) were isolated by gradient centrifugation, frozen in 10% DMSO medium and stored in liquid N2 until analysis. Before analysis, the cells were thawed quickly in a 37 °C water bath until a tiny ice crystal remained, then diluted slowly with warm media containing DNase I (to prevent DNA-induced clumping), washed twice, and left for 2 h at RT in 10% RPMI before staining.

### 4.3. Multicolor Phenotyping Analysis of Surface Markers

Two multiparameter flow cytometry panels were used to identify T-cell subpopulations. Panel 1 was designed with the following surface markers: CD3-BUV395 (clone UCHT1, cat# 563548, BD Horizon, Franklin Lakes, NJ, USA), CD4-BUV496 (clone SK3, cat# 612936, BD Horizon), CD15s-BV510 (clone CSLEX1, cat# 563529, BD Horizon), CD25-BV421 (clone M-A251, cat# 356114, BioLegend, San Diego, CA, USA), CD31-BV605 (clone WM59, cat# 562855, BD Horizon), CD45RA-PE-Cy7 (clone L48, cat# 337186, BD), CD127-BB700 (clone HIL-7R-M21, cat# 566396, BD Horizon), CD161-PE (clone DX12, cat# 556081, BD Pharmingen, San Diego, CA, USA), CD183-BV711 (clone 1C6/CXCR3, cat# 563156, BD Horizon), CD185-BB515 (clone RF8B2, cat# 564624, BD Horizon), CD194-BV786 (clone 1G1, cat# 744141, BD OptiBuild, Franklin Lakes, NJ, USA), CD196-R718 (clone 11A9, cat# 752203, BD OptiBuild), CCR10-APC (clone 1B5, cat# 564711, BD Pharmingen).

Panel 2 included: CD3-BUV395 (clone UCHT1, cat# 563548, BD Horizon), CD4-BUV496 (clone SK3, cat# 612936, BD Horizon), CD15s-BV510 (clone CSLEX1, cat# 563529, BD Horizon), CD25-BV421 (clone M-A251, cat# 356114, BioLegend), CD38-BV650 (clone HB-7, cat# 356620, BioLegend), CD39-BV711 (clone TU66, cat# 563680, BD Horizon), CD45RA-PE-Cy7 (clone L48, cat# 337186, BD), CD71-FITC (clone M-A712, cat# 555536, BD Pharmingen), CD73-PE-CF594 (clone AD2, cat# 562817, BD Horizon), CD127-BB700 (clone HIL-7R-M21, cat# 566396, BD Horizon), CD147-BV786 (clone HIM6, cat# 742630, BD OptiBuild), CD161-PE (clone DX12, cat# 556081, BD Pharmingen), GARP-APC (clone 7B11, cat# 562341, BD Pharmingen), HLA-DR-R718 (clone G46-6, cat# 567035, BD Horizon), PI16-BV605 (clone CRCBT-02-001, cat# 745153, BD OptiBuild).

In addition, CD4-BUV496 (clone SK3, cat# 612936, BD Horizon), CD8a-BV510 (clone RPA-T8, cat# 301048, BioLegend), CD62L-PE-Cy7 (clone DREG-56, cat# 304822, BioLegend), CD45RA-BV605 (clone HI100, cat# 304134, BioLegend), and CD95-AF488 (clone DX2, cat#305616, BioLegend) were used to characterize CD4 and CD8 naïve, central memory, effector memory and terminally differentiated effector memory subsets.

Samples were stained by adding pre-titrated concentrations of directly conjugated antibodies suspended in staining buffer (Brilliant Stain Buffer Plus, cat# 566385, BD Horizon, San Jose, CA, USA), followed by a 15 min incubation at room temperature in the dark, and washed with PBS (cat# TMS-012, Merck KGaA, Darmstadt, Germany). A viability dye—DAPI Solution (cat# BDB564907, BD Biosciences, San Jose, CA, USA)—at a concentration of 0.5 µg/mL was added 2 to 5 min before acquiring the sample on FACS Aria Fusion (equipped with blue, red, violet and UV lasers, BD Biosciences) and analyzed with FACS Diva v. 9.0.1 (BD Biosciences, San Jose, CA, USA) and FlowJo software v.10.10.0 (FlowJo, LLC, Ashland, OR, USA). At least 5 000 Treg events were acquired. Fluorescence Minus One (FMO) controls were used for accurate gating of positive populations. Cytometer Setup & Tracking Beads (BD CST, cat # 642412) were used before sample acquisition.

Since analyses were performed at the two recruitment sites, the following conditions were ensured to minimize bias in analysis: similar flow cytometers (FACSARia III), identical working and cleaning solutions, calibration beads, daily maintenance procedures, and SOPs for processing and immunophenotyping of blood samples were employed. All monoclonal antibodies included in panel 1 and panel 2 were obtained through the same tender procedure in double quantities and shared between the centers. Preliminary titration of each antibody was performed to determine the optimal working concentration, maximizing the signal-to-noise ratio. After calibration and test runs of the panels, instrument settings and analysis results were shared to ensure the consistency of results. Daily calibration was performed to maintain stable laser and detector settings; the same antibody reagents (manufacturer, lot numbers) and working concentrations were used throughout the study; a uniform gating strategy across all data files was applied to ensure that data remained directly comparable. Operators at the two sites were blinded to the acquired samples. The analysis of acquired data (FloJo) was performed entirely in Sofia to eliminate additional sources of variation.

#### Gating Strategy for Treg and Effector Th Subsets

Time vs. FSC was plotted to check for fluidic instability or pressure drops; a FSC-A vs. SSC-A plot was used to exclude the heavy debris cloud in the bottom-left corner; singlets were isolated on a FSC-H vs. FSC-A plot, and the viability dye-stained cells were excluded. Subsequently, lymphocytes were defined on a FSC vs. SSC plot, and CD4 T cells were further identified as CD3+CD4+ events. Regulatory T cells (Tregs) and conventional T cells (non-Tregs) were identified based on differential expression of CD127 and CD25. From the non-Treg gate, antigen-primed T and T follicular helper (Tfh) cells were defined as CD45RA–CD185– and CD45RA–CD185+ cells, respectively. Tfh cells were further dissected into CD183+CD196– Tfh1, CD183–CD196– Tfh2 and CD183–CD196+ Tfh17 subsets. CD45RA- T cells were further dissected into Th1 (CD183+CD194–CD196–), Th2 (CD183–CD194+CD196–CCR10–), Th17 (CD183–CD194+CD196+CCR10–), Th22 (CD183–CD194+CD196+CCR10+), Th9 (CD194–CD196+) and ThG (CD183–CD194+CD196–CCR10+) subsets. Within the Treg gate, T follicular regulatory cells (Tfr) were defined as CD45RA–CD185+ cells, while CD45RA-Tregs were further dissected into Th1-like (CD183+ CD196–), Th2-like (CD183–CD196–), Th17-like (CD183+/–CD196+CD194+CCR10–) and Th22-like (CD183+/–CD196+CD194+CCR10+) subsets (Supplementary Figure S1).

According to the expression of CD45RA, Tregs were defined as naïve (CD45RA+) and effector (CD45RA-) Tregs, respectively. Fluorescence Minus One (FMO) controls were used for setting gates on markers with continuous expression (CD38, CD39, CD71, CD73, CD147, GARP, HLA-DR and PI16) on both naïve (nTreg) and effector Tregs (eTreg). Within the eTreg gate, the discrete subsets of CD15s+ and CD161 subsets were identified, which were previously characterized as highly activated and inflammatory cytokine-producing, respectively. Activation of non-Tregs was determined based on the expression of CD38 and HLA-DR (Supplementary Figure S2).

From the lymphocyte gate, CD4 and CD8 T cells were identified as CD3+CD4+ and CD3+CD8+ events and further dissected into CD45RA+CD62L+naïve (N), CD45RA–CD62L+ central memory (CM), CD45RA–CD62L– effector memory (EM), and CD45RA+CD62L– terminally differentiated effector (TE) subsets.

### 4.4. Serum Biomarker Assessment

The following biochemical and immunological markers were measured using enzyme-linked immunosorbent assay (ELISA), according to the manufacturer’s instructions: catalase (2–600 kU/L, BT LAB, Jiaxing, Zhejiang, China, cat# E3053Hu), superoxide dismutase 1 (SOD1, 0.5–180 ng/mL, BT LAB, Jiaxing, Zhejiang, China, cat# E4502Hu), glutathione peroxidase 1 (GPX1, 31.25–2000 pg/mL, Elabscience, Wuhan, Hubei, China, cat# E-EL-H5410), malondialdehyde (31.25–2000 ng/mL, Elabscience/Wuhan, Hubei, China, cat# E-EL-0060), nitric oxide synthase 1 (NOS1, 0.156–10 ng/mL, USCN/SEA815Hu), nitric oxide synthase 3 (NOS3, 62.5–4000 pg/mL, Elabscience, Wuhan, Hubei, China, cat# E-EL-H0755), zonulin (2–600 ug/mL, BT LAB, Jiaxing, Zhejiang, China, cat# E1117Hu), lipopolysaccharide (LPS, 0.47–30 ng/mL, USCN, Houston, TX, USA, cat# USEB526Hu), immunoglobulin G (IgG) against lipopolysaccharide (3.12–200 ng/mL, USCN/Houston, USA, cat# AEB526Ge), Toll-like receptor 4 (TLR4, 0.05–15 ng/mL ng/mL, BT LAB/Jiaxing, Zhejiang, China cat# E0346Hu), indoleamine 2,3-dioxygenase (IDO, 0.3–90 ng/mL, BT LAB/Jiaxing, Zhejiang Province, cat# E0796Hu), and Dickkopf-related protein 1 (DKK1, 0.5–150 ng/mL, BT LAB/Jiaxing, Zhejiang Province, China, cat# E0630Hu).

### 4.5. Clinical Examination

To assess the subjective severity of symptoms, all participants completed the following standardized and validated scales.

#### 4.5.1. FibroFatigue Scale (FFS)

It includes 12 clinical parameters covering pain-related, autonomic, and cognitive complaints. Each item is rated from 0 to 6, with higher scores indicating more severe symptom expression [67].

#### 4.5.2. Hamilton Anxiety Rating Scale (HAMA-14)

It measures anxiety across two domain subscales: Psychic Anxiety (psychological symptoms such as tension, fear, insomnia, and depressive mood) and Somatic Anxiety (somatic manifestations including muscle tension, autonomic, and gastrointestinal symptoms). The total score is calculated as the sum of 14 items [30].

#### 4.5.3. Hamilton Depression Rating Scale (HAMD-17)

It assesses the severity of depressive symptoms and associated somatic manifestations, including subscales for depressed mood, guilt, insomnia, anxiety, and somatic and gastrointestinal symptoms. It was used as a tool for excluding severe affective disorders and for stratification according to the depressive component [30].

#### 4.5.4. DePaul Symptom Questionnaire

This self-report tool assesses and screens for myalgic encephalomyelitis/chronic fatigue syndrome (ME/CFS) and related post-viral conditions [68].

In addition, refined scores focusing on core symptom domains (pHAMA, pHAMD, and pFF) were considered in the composite evaluation to reduce the influence of overlapping somatic features, in accordance with previously published approaches for symptom domain separation in post-infectious and chronic fatigue-related conditions [15,30]. Two subdomains of the HAMD were used; the first was pure depressive symptoms (pure HAMD), which was the sum of sad mood, feelings of guilt, suicidal thoughts, and loss of interest; the second was physio-somatic HAMD (physiosom HAMD), which was the sum of somatic anxiety, gastrointestinal (GIS) anxiety, genitourinary anxiety, and hypochondriasis. Likewise, two subdomains of the HAMA were computed: namely, pure anxiety symptoms (pure HAMA), the sum of anxious mood, tension, fears, anxiety, and anxious behavior during the interview, and physio-somatic HAMA symptoms (physiosom HAMA), the sum of somatic sensory, cardiovascular, genitourinary, and autonomic symptoms, as well as GIS. Furthermore, after omitting items reflecting cognitive and affective symptoms in the FF scale, a pure physio-somatic FF (pure FF) score was computed as the sum of muscular pain, muscle tension, fatigue, autonomous symptoms, GIS, headache, and flu-like malaise [30,69,70]. The derived psychometric variables were subsequently used in statistical analyses, including correlation analysis and age- and sex-adjusted ANCOVA models, in order to assess their association with biochemical parameters and to evaluate differences between subgroups and healthy controls.

### 4.6. Statistical Analysis

Statistical analyses were performed using IBM SPSS Statistics version 23.0 (IBM Corp., Armonk, NY, USA). Graphical representations were generated using GraphPad Prism version 9.0 (GraphPad Software, San Diego, CA, USA). The normality of data distribution was assessed using the Shapiro–Wilk test. Continuous baseline variables were compared using one-way ANOVA or appropriate/nonparametric alternatives when assumptions were not adequately met. Categorical variables were compared using chi-square tests where applicable. To control for multiple comparisons, the Benjamini–Hochberg false discovery rate (FDR) procedure was applied. Markers surviving FDR correction were further evaluated using age/BMI/sex-adjusted ANCOVA models to analyze covariance. Associations between variables were assessed using the Spearman’s rank correlation test. Nominal correlations that did not survive FDR correction were interpreted as exploratory only.

Finally, Random Forest classification was used as an exploratory machine learning approach followed by logistic regression analyses to evaluate the classification potential and independent contribution of selected biomarkers.

The RF classifier consisted of 150 decision trees and used the Gini impurity criterion for node splitting. Balanced class weights were applied to account for unequal group sizes.

Three pairwise binary classification models were fitted: LC vs. HC, ME/CFS vs. HC, and LC vs. ME/CFS. In addition, a three-class RF model was fitted to simultaneously discriminate among LC, ME/CFS, and HC participants. For the binary models, the positive class used for the calculation of sensitivity was defined as LC for the LC vs. HC comparison, ME/CFS for the ME/CFS vs. HC comparison, and ME/CFS for the LC vs. ME/CFS comparison.

Model performance was evaluated using a nested cross-validation framework to minimize optimism arising from model selection and hyperparameter tuning. In the outer loop, stratified five-fold cross-validation was repeated twice using different fold assignments, resulting in 10 held-out test folds for each pairwise comparison and for the three-class model. Stratification was used to preserve the relative distribution of the outcome classes across the cross-validation folds.

Within each outer training set, hyperparameter optimization was performed using stratified three-fold cross-validation. The hyperparameters evaluated were the number of features considered at each split (max_features: 1, 2, or 3) and the minimum number of observations required in a terminal node (min_samples_leaf: 1, 3, or 6). Hyperparameter combinations were selected according to the highest mean area under the receiver operating characteristic curve (AUC) obtained during the inner cross-validation. For the three-class model, performance during hyperparameter selection was assessed using macro-averaged one-vs-rest AUC.

Missing data were handled using multiple imputation, with five imputed datasets. To prevent information leakage, the imputation model was fitted separately within each training subset of the corresponding cross-validation split. During the inner cross-validation procedure, the imputer was refitted within each inner training split and subsequently applied to the corresponding validation split. Thus, information from validation or test observations was not used to estimate imputation parameters.

Following hyperparameter selection, a separate RF model was fitted for each of the five imputed datasets using the complete outer training set and the hyperparameters selected in the inner cross-validation. The outer test fold was then imputed using the imputation model fitted exclusively to the corresponding outer training set, and predictions were generated for the held-out observations. Predicted class probabilities were subsequently averaged across the five imputed datasets to obtain pooled predictions for each participant (prediction pooling).

The pooled predicted probabilities were used for the primary calculation of classification performance, while the variability of AUC estimates across the five imputations was reported separately to characterize the sensitivity of model performance to the imputation procedure (Supplementary Table S2).

This nested cross-validation and prediction-pooling framework ensured that hyperparameter selection, imputation, model fitting, and performance estimation were conducted without information leakage from the held-out test observations.

Logistic regression was used to identify which Random Forest-prioritized biomarkers retained independent linear associations with group status. A binary logistic regression was performed with the two most complete datasets, MDA and GPX1, without any imputations.

Missing data were handled according to the specific analysis. For one-way ANOVA and ANCOVA, cases were included when valid data were available for the dependent variable and model variables. For Spearman correlations, pairwise deletion was used. Logistic regression analyses used complete cases for the predictors included in each model.

## 5. Conclusions

This cross-sectional study on well-defined LC, ME/CFS and HC groups identified five serum and T-cell biomarkers: MDA, GPX1, Th17, CD15s+CD161−eTreg, and CD161+eTreg that differentiated between the groups, independent of age, BMI and sex. ME/CFS patients displayed significantly elevated MDA and GPX1, while LC patients were distinguished by a significant decrease in Th17 at the expense of CD15s+CD161−eTreg, regulatory (GARP+nTreg) and effector (CD38-HLA-DR+ CD4, Tfh17) subsets, which correlated with serum biomarkers of oxidative stress (Ig-LPS, NOS3). However, no immune–mental health correlations were established. In exploratory Random Forest models, MDA and GPX1 accurately discriminated ME/CFS from both HC and LC. A possible interpretation of the differential biomarkers’ expression in the syndrome groups is that ME/CFS and LC differ biochemically in the extent of cellular damage and oxidative stress compensation capacity.

## 6. Strengths and Limitations of the Study

The study addresses a significant and unsolved problem: identification of phenotypic cellular/serum biomarkers with diagnostic potential. The study groups were well defined and evaluated by medical doctors. A comprehensive analysis of effector and regulatory T-cell subsets was performed, combined with detailed questionnaires for clinical evaluation.

At the same time, a number of limitations should be acknowledged:

The cross-sectional design limits conclusions regarding temporal dynamics and causality. The sample size was moderate, particularly within subgroup analyses, and may limit statistical power for detecting smaller effects. The eligibility criteria applied to healthy controls were very stringent and may have increased the observed differences between the control group and the patient groups. An additional limitation is that the COVID-19 vaccination status of participants was not included in the analysis. Despite the fact that participants in the ME/CFS group had no reported history of SARS-CoV-2 infection, and in most cases their symptoms preceded the pandemic, vaccination-related immune effects cannot be completely excluded. Analyses were entirely phenotypic and restricted to peripheral blood, and thus may not fully reflect functional or tissue-specific immune alterations. Freeze-thawing of PBMCs may have altered the expressions of some surface markers. Information on smoking status was not collected, though it can influence oxidative-stress biomarkers. Altogether, the findings of the study should be interpreted as exploratory and hypothesis-generating.

## Figures and Tables

**Table 1:** Demographic and medical characteristics of participants.

Variable/Group	LC	ME/CFS	HC	*p* Value
Samples (*n*)	89	125	80	
Sex M (*n*/%)	25/28.1	33/26.4	27/33.8	*p* = 0.52
Sex F (*n*/%)	64/71.9	92/73.6	53/66.2	
Age (mean ± SD) Median [Q1–Q3]	48.67 ± 11.81 48.0 [39.5–58.8]	44.84 ± 13.06 44.0 [36.0–55.0]	30.28 ± 12.99 26.0 [22.0–43.0]	*p* < 0.001
BMI (mean ± SD)	24.96 ± 4.81	24.66 ± 5.15	22.51 ± 4.64	*p* = 0.015 (LC vs. HC) *p* = 0.028 (ME/CFS vs. HC)
Disease duration * (months) Median [Q1–Q3]	24 [16.0–30.0]	36 [6.0–84.0]	na	*p* < 0.0001
Reported co-morbidities	1/89	7/125	0/80	*p* = 0.089
Reported current medication	2/89	2/125	0/80	*p* = 0.432
COVID-19 Vaccination	7/89	19/125	22/80	*p* <0.01

* For LC: time since positive test result; for ME/CFS: time since symptoms onset. BMI = body mass index, LC = long COVID, ME/CFS = myalgic encephalomyelitis/chronic fatigue syndrome, HC = healthy controls.

**Table 2:** Unadjusted and age/BMI/sex-adjusted ANCOVA results for FDR-prioritized biomarkers.

Marker	Raw *p*	Age F	Age *p*	Age Partial η^2^	BMI F	BMI *p*	BMI Partial η^2^	Sex F	Sex *p*	Sex Partial η^2^
**MDA**	0.000	4.549	**0.018**	0.211	5.681	**0.007**	0.250	5.717	**0.007**	0.252
IgG-LPS	0.000	2.845	0.072	0.143	3.068	0.060	0.153	3.307	**0.049**	0.163
**NOS3**	0.000	4.417	**0.020**	0.206	4.513	**0.018**	0.210	4.284	**0.022**	0.201
**GPX1**	0.000	3.457	**0.043**	0.169	4.184	**0.024**	0.198	4.056	**0.026**	0.193
CAT	0.001	0.239	0.789	0.014	0.249	0.781	0.014	0.367	0.696	0.021
SOD1	0.013	2.817	0.074	0.142	2.717	0.080	0.138	3.233	0.052	0.160
TLR4	0.000	0.799	0.458	0.045	0.715	0.496	0.040	0.766	0.473	0.043
ZONULIN	0.000	2.338	0.112	0.121	2.398	0.106	0.124	2.331	0.113	0.121
**Th17**	0.004	5.749	**0.007**	0.253	5.402	**0.009**	0.241	5.336	**0.010**	0.239
CD38−HLA-DR+	0.000	1.745	0.190	0.093	1.981	0.153	0.104	1.644	0.208	0.088
CD38+HLA-DR-	0.004	1.482	0.241	0.080	0.556	0.579	0.032	0.825	0.447	0.046
CD31+eTreg	0.013	1.793	0.182	0.095	0.979	0.386	0.054	0.825	0.447	0.046
**CD161+eTreg**	0.004	5.806	**0.007**	0.255	7.113	**0.003**	0.295	5.885	**0.006**	0.257
GARP+eTreg	0.004	0.913	0.411	0.051	0.900	0.416	0.050	1.335	0.277	0.073
**CD15s+CD161−eTreg**	0.000	13.905	**0.000**	0.450	14.139	**0.000**	0.454	14.986	**0.000**	0.469
CD4+CD95+ *	0.001	9.896	**0.002**	0.055	0.649	0.422	0.004	0.225	0.636	0.001

* Unadjusted differences (raw *p*) identified by Kruskal–Wallis/one-way ANOVA, BMI = body mass index, MDA = malondialdehyde, IgG-LPS = immunoglobulin G against lipopolysaccharide, NOS3 = nitric oxide synthase, GPX1 = glutathione peroxidase 1, CAT = catalase, SOD1 = superoxide dismutase, TLR4 = toll-like receptor 4, zonulin Th17 = T helper cells 17, eTregs = effector regulatory T cells. Statistically significant differences between groups and the corresponding markers are bolded.

**Table 3:** Pairwise comparison of age/BMI-adjusted ANCOVA biomarkers.

Marker	LC vs. ME/CFS				LC vs. HC				ME/CFS vs. HC			
	Age *p*	Age adj. *p*	BMI *p*	BMI adj. *p*	Age *p*	Age adj. *p*	BMI *p*	BMI adj. *p*	Age *p*	Age adj. *p*	BMI *p*	BMI adj. *p*
MDA	0.17	0.52	0.13	0.40	0.24	0.72	0.19	0.56	0.01	0.02	0.00	0.01
NOS3	0.40	1.00	0.44	1.00	0.01	0.03	0.01	0.03	0.02	0.07	0.02	0.06
GPX1	0.50	1.00	0.40	1.00	0.14	0.41	0.12	0.35	0.01	0.04	0.01	0.02
Th17	0.14	0.41	0.13	0.38	0.00	0.01	0.00	0.01	0.03	0.08	0.04	0.11
CD161+eTreg	0.13	0.38	0.06	0.18	0.19	0.56	0.21	0.62	0.00	0.01	0.00	0.00
CD15s+CD161−eTreg	0.05	0.14	0.03	0.10	0.00	0.00	0.00	0.00	0.00	0.00	0.00	0.00

BMI = body mass index, MDA = malondialdehyde, NOS3 = nitric oxide synthase, GPX1 = glutathione peroxidase 1, Th17= T helper cells 17, eTregs = effector regulatory T cells, LC = long COVID, ME/CFS = myalgic encephalomyelitis/chronic fatigue syndrome, HC = healthy controls.

**Table 4:** (**A**) Age-restricted comparison of ANCOVA surviving biomarkers (Kruskal–Wallis). (**B**) BMI-restricted comparison of ANCOVA surviving biomarkers (Kruskal-Wallis).

(**A**)							
		**Age restr. *p***			**Age restr. adj. *p***		
**Biomarker**	**Age group restr. *p***	**LC vs. ME/CFS**	**LC vs. HC**	**ME/CFS vs. HC**	**LC vs. ME/CFS**	**LC vs. HC**	**ME/CFS vs. HC**
MDA	0.000	0.000	0.039	0.000	0.000	0.161	0.000
NOS3	0.530	0.949	0.289	0.290	0.997	0.456	0.456
GPX1	0.000	0.000	0.122	0.000	0.000	0.366	0.000
Th17	0.021	0.056	0.008	0.200	0.169	0.024	0.599
CD161+eTreg	0.003	0.650	0.005	0.001	1.000	0.015	0.003
CD15s+CD161−eTreg	0.003	0.005	0.003	0.274	0.016	0.008	0.821
(**B**)							
		**BMI restr. *p***			**BMI restr. adj. *p***		
**Biomarker**	**BMI group restr. *p***	**LC vs. ME/CFS**	**LC vs. HC**	**ME/CFS vs. HC**	**LC vs. ME/CFS**	**LC vs. HC**	**ME/CFS vs. HC**
MDA	0.000	0.000	0.067	0.000	0.000	0.202	0.000
NOS3	0.000	0.803	0.001	0.000	1.000	0.003	0.001
GPX1	0.000	0.000	0.143	0.000	0.000	0.430	0.000
Th17	0.000	0.084	0.000	0.006	0.254	0.000	0.017
CD161+eTreg	0.051	0.465	0.090	0.015	0.326	0.094	0.031
CD15s+CD161−eTreg	0.000	0.012	0.000	0.012	0.036	0.000	0.037

BMI = body mass index, MDA = malondialdehyde, NOS3 = nitric oxide synthase, GPX1 = glutathione peroxidase 1, Th17 = T helper cells 17, eTregs = effector regulatory T cells, LC = long COVID, ME/CFS = myalgic encephalomyelitis/chronic fatigue syndrome, HC = healthy controls.

**Table 5:** Nominal immune–mental health correlations before FDR correction.

Cohort	Immune Marker	Mental-Health Variable	Spearman rho	Raw *p*	FDR q
Whole cohort	MDA	DePol-Tot	0.218	0.029	0.480
Whole cohort	MDA	PS-HAMA	0.199	0.032	0.480
LC + ME/CFS	IgG-LPS	T-FF	−0.300	0.002	0.060
LC + ME/CFS	IgG-LPS	Pu-FF	−0.234	0.019	0.285
LC + ME/CFS	IgG-LPS	T-HAMA	−0.205	0.041	0.410

LC = long COVID, ME/CFS = myalgic encephalomyelitis/chronic fatigue syndrome, HC = healthy controls, MDA = malondialdehyde, IgG-LPS = immunoglobulin G against lipopolysaccharide, DePol-Tot = total depolarization score, PS-HAMA = somatic Hamilton anxiety rating score, T-FF = total FibroFatigue score, Pu-FF = pure FibroFatigue score, T-HAMA = total Hamilton anxiety rating score.

**Table 6:** Top Random Forest predictors by pairwise comparison.

Comparison	Rank 1	Rank 2	Rank 3
LC vs. HC	MDA	CD15s+CD161−eTreg	Th17
ME/CFS vs. HC	MDA	GPX1	CD161+eTreg
ME/CFS vs. LC	GPX1	MDA	CD15s+CD161−eTreg

LC = long COVID, ME/CFS = myalgic encephalomyelitis/chronic fatigue syndrome, HC = healthy controls, MDA = malondialdehyde, GPX1 = glutathione peroxidase 1, eTregs = effector regulatory T cells, Th17 = T helper cells 17.

**Table 7:** Logistic regression model performance summary.

Model	Positive Class	χ^2^	df	*p*-Value	Nagelkerke R^2^	Hosmer–Lemeshow *p*	Correct Classification	AUC	95% CI for AUC
LC vs. HC	LC	12.65	2	0.002	0.111	<0.0001	57.4%	0.577	0.492–0.659
ME/CFS vs. HC	ME/CFS	123.38	2	<0.001	0.670	0.061	86.1%	0.924	0.883–0.958
LC vs. ME/CFS	ME/CFS	50.82	2	<0.001	0.342	0.104	75.9%	0.777	0.702–0.849

LC = long COVID, ME/CFS = myalgic encephalomyelitis/chronic fatigue syndrome, HC = healthy controls.

## Data Availability

Requests for access to the study data can be submitted to the corresponding author.

## Supplementary Materials

The following supporting information can be downloaded at https://www.mdpi.com/article/10.3390/ijms27219422/s1.

## Abbreviations

The following abbreviations are used in this manuscript:

ME/CFS	Myalgic encephalomyelitis/chronic fatigue syndrome
COVID	Coronavirus infectious disease
LC	Long coronavirus infectious disease
SARS-CoV-2	Severe acute respiratory syndrome coronavirus 2
ELISA	Enzyme-linked immunosorbent assay
HAMA	Hamilton anxiety rating scale
HAMD	Hamilton depression rating scale
FF	Fibro fatigue
PEM	Post-exertional malaise
POTS	Postural orthostatic tachycardia syndrome
CD	Cluster of differentiation
NK	Natural killer
PBMC	Peripheral blood mononuclear cell
Th	T helper cell
Tfh	T follicular helper cell
Treg	T regulatory cell
Tfr	T follicular regulatory cell
IL	Interleukin
ANS	Autonomic nervous system
HPA	Hypothalamic–pituitary–adrenal
HIV	Human immunodeficiency virus
PI	Peptidase inhibitor
GARP	Glycoprotein A repetitions predominant
IOM	Institute of Medicine
HC	Healthy controls
IgG	Immunoglobulin G
TUD	Tobacco use disorder
SSRIs	Selective serotonin reuptake inhibitors
DMSO	Dimethyl sulfoxide
DNase	Deoxyribonuclease
FSC	Forward scatter
SSC	Side scatter
HLA	Human leukocyte antigen
CCR	CC chemokine receptor
nTregs	Naïve T regulatory cells
eTregs	Effector T regulatory cells
CAT	Catalase
SOD	Superoxide dismutase
GPX	Glutathione peroxidase
NOS	Nitric oxide synthase
MDA	Malondialdehyde
LPS	Lipopolysaccharide
TLR	Toll-like receptor 4
IDO	Indoleamine 2,3-dioxygenase
DKK1	Dickkopf-related protein 1
FFS	FibroFatigue scale
qSUM	Combined questionnaire score
ANOVA	Analysis of variance
ANCOVA	Analysis of covariance
FDR	False discovery rate
N	Naïve
CM	Central memory
EM	Effector memory
TE	Terminal effector
IQR	Interquartile range
