# Supplementary material for: Treg and Oxidative Stress Biomarker Profiles in Long COVID and Myalgic Encephalomyelitis/Chronic Fatigue Syndrome
Source: Int J Mol Sci. 2026 Oct 22;27(21):9422. doi: 10.3390/ijms27219422 (PMC13763008; doi:10.3390/ijms27219422)
Supplement: Supplementary file 1 [file ijms-27-09422-s001.zip › Information for the study.pdf]

INFORMATION ABOUT THE STUDY

Study topic: BIOMARKERS IN POST-COVID AND CHRONIC FATIGUE SYNDROME

The study is organized by the Department of Medical Microbiology and Immunology "Prof. Dr. Elisey Yanev" and SIMU of the Medical University-Plovdiv within the framework of the project " *Innovative diagnostic methods for detecting congenital and acquired immune deficiencies* " under the " *Program for Strategic Research and Innovation for the Development of MU - Plovdiv (PSRID-MUP)* "

Duration of the study : 2023-2026.

The main goal of the project is to study a set of biomarkers (laboratory indicators) related to clarifying the causes of post-COVID and chronic fatigue syndrome and their diagnosis and monitoring. This will help to develop medical knowledge about these diseases and improve their diagnosis and monitoring of treatment.

The study will include: individuals with **post-COVID symptoms** or **Chronic Fatigue Syndrome**

- The selection of participants will be random, and men and women between the ages of 18 and 65 will be included.
- The study will include 100 individuals with post-COVID and 100 individuals with Chronic Fatigue Syndrome.

The following will be excluded from the study: pregnant women, nursing mothers, and individuals with immune deficiency, diabetes, systemic autoimmune and allergic diseases, neurodegenerative and mental illnesses, etc.).

The study will be conducted in **two locations** :

- **The Department of Medical Microbiology and Immunology "Prof. Dr. Elisey Yanev" - and the SIMU of Medical University-Plovdiv** : tel. 0877 842 487 and email CFS.Bulgaria@gmail.com
- **National Institute of Infectious and Parasitic Diseases-Sofia** : tel. 02 943 56 36 and email imlab @ ncipd . org

After reviewing the study information and obtaining informed consent to participate, **the study will include the following**:

1. Assessment of the severity of symptoms characteristic of chronic fatigue syndrome using four scales.
2. Collection of venous blood in a volume of 18 ml with a closed system in three tubes and 2 ml in a serum tube.
3. The participant will be asked to provide a stool sample for gut microbiome testing.
4. A portion of the blood will be tested for differential blood count, absolute T-B- NK cell count and percentage, and cytokine profile. The results of these tests will be provided to the participant within 2-3 days.
5. Serum, plasma, and lymphocytes will be isolated from the remaining blood and stored frozen until their analysis.
6. Serum and plasma will be tested for cytokine profile, latent viral infections (Herp-6, EBV, CMV, SARS-Cov-2); autoantibodies; oxidative and nitrosative stress; intestinal permeability and neurofilaments.

7. Lymphocytes will be analysed for phenotype and functions of T-cell subpopulations and NK cells.
8. The intestinal microbiome in the stool will be examined for the presence of dysbiosis.

If necessary, the participant will be referred to other specialists and additional tests.

Confidentiality. All participants will be included in the study under a unique number. Confidentiality will be maintained throughout the study by the research team responsible for labeling/labeling the questionnaires and samples.
