# Supplementary material for: Treg and Oxidative Stress Biomarker Profiles in Long COVID and Myalgic Encephalomyelitis/Chronic Fatigue Syndrome
Source: Int J Mol Sci. 2026 Oct 22;27(21):9422. doi: 10.3390/ijms27219422 (PMC13763008; doi:10.3390/ijms27219422)
Supplement: Supplementary file 1 [file ijms-27-09422-s001.zip › Patient Cart.pdf]

PATIENT CART

1. **Demographics:**
   - Name…………………………………………………Code………..
   - Date of birth…………………………………… Gender M/F
   - Height……cm Weight………..kg
   - Place of residence: ……………………..
   - Telephone…………………………………………………………
   - E-mail……………………………………………………………
2. **For post-COVID patients** –
   a. date of positive test for COVID-19 (PCR/ antigen) :…………………
   b. Severity: outpatient, in hospital

OUTPATIENT	IN THE HOSPITAL
	Medium – without oxygen
	Heavy – with oxygen
	Very serious – in intensive care unit

3. **For patients with CFS** – date of onset of symptoms:………………................

   Past COVID-19: NO YES - Date

4. **Collection of biological material : date** ………………………
   - 18ml. venous blood in vacutainers / monovets with sodium heparin
   - 2ml venous blood in a vacutainer/monovet for serum
   - Feces Date………
